# Supplementary material for: Cross-scale interaction of host tree size and climatic water deficit governs bark beetle-induced tree mortality
Source: Nat Commun. 2021 Jan 8;12:129. doi: 10.1038/s41467-020-20455-y (PMC7794511; doi:10.1038/s41467-020-20455-y)
Supplement: Supplementary file 1 — Supplementary Information [file 41467_2020_20455_MOESM1_ESM.pdf]

Supplementary Information

Date report generated: November 19, 2020

Supplementary Notes

1. Basal area consequences of proportion of host trees killed

An increasing proportion of dead host trees corresponded to increasing proportion of host basal area killed and total basal area killed (Supplementary Figure 1). Coupled with the positive effect of mean host tree height on the probability of host tree mortality, we suggest that our model coefficient estimates can also be interpreted as the relative influence on the proportion of host basal area killed.

2. Effect of Level 3b tree height calibration on model results

Here, we show the original fitted model prior to calibrating the tree heights as detailed above. Comparing this figure of posterior distributions of model parameters with the fitted model we report in the main paper, we find that the height calibration strengthens the CWD effect and reverses the host tree height effect. Supplementary Figure 3 shows a negative effect of host tree mean height on host mortality, while we report in the main text (using the calibrated heights) a positive main effect of host tree mean height.

Supplementary Tables

Supplementary Table 1: Site characteristics for each of the 32 sites. The site name consists of the forest name, elevation band, and rep separated by an underscore. The Eldorado National Forest is ‘eldo’, the Stanislaus National Forest is ‘stan’, the Sierra National Forest is ‘sier’, and the Sequoia National Forest is ‘sequ’. The elevation band represents the lower bounds of the 305 meter (1000 foot) elevation bands in feet. Thus ‘3k’ implies that site was located between 3,000 and 4,000 feet (914-1219 meters). Aerially detected mortality and density of the whole site is presented along with the mortality and density calculated from the ground data (aerial / ground). The density is measured in trees per hectare (tpha).

Site	CWD (mm)	CWD (z-score)	Survey area (ha)	Overall density (tpha; aerial/ground)	Mortality density (tpha; aerial/ground)	% tree mortality (aerial/ground)
eldo_3k_1	678	0.319	31.02	630/410	69/252	10.9/61.4
eldo_3k_2	706	0.501	30.61	444/647	51/232	11.5/35.9
eldo_3k_3	655	0.163	30.95	493/410	108/148	21.9/36.1
eldo_4k_1	570	-0.383	28.04	633/588	55/232	8.7/39.5
eldo_4k_2	642	0.0831	28.41	338/272	51/213	15.2/78.2
eldo_5k_1	663	0.219	28.44	662/544	75/237	11.4/43.6
eldo_5k_2	627	-0.0132	30.02	585/969	73/351	12.5/36.2
eldo_5k_3	599	-0.2	29.73	489/623	33/198	6.8/31.7
stan_3k_1	638	0.059	31.04	739/1038	73/544	9.9/52.4
stan_3k_2	739	0.713	18.78	434/405	175/316	40.4/78.0
stan_3k_3	762	0.859	30.1	558/326	121/133	21.6/40.9
stan_4k_1	540	-0.58	29.62	508/712	148/450	29.2/63.2
stan_4k_2	528	-0.658	30.54	482/257	88/143	18.3/55.8
stan_5k_1	524	-0.688	30.94	389/336	75/183	19.3/54.4
stan_5k_2	524	-0.685	29.94	399/623	85/272	21.3/43.7
sier_3k_1	764	0.871	30.42	651/850	121/405	18.6/47.7
sier_3k_2	768	0.898	30.05	439/153	89/119	20.3/77.4
sier_3k_3	773	0.932	29.77	511/460	161/356	31.5/77.4
sier_4k_1	841	1.38	30.43	576/539	309/277	53.6/51.4
sier_4k_2	764	0.877	29.3	499/855	163/489	32.6/57.2
sier_4k_3	688	0.383	26.39	454/499	218/297	48.1/59.4
sier_5k_1	722	0.599	14.59	631/717	258/311	40.9/43.4
sier_5k_2	710	0.523	27.53	477/455	252/336	52.9/73.9

Site	CWD (mm)	CWD (z-score)	Survey area (ha)	Overall density (tpha; aerial/ground)	Mortality density (tpha; aerial/ground)	% tree mortality (aerial/ground)
sier_5k_3	779	0.968	28.93	569/484	186/208	32.6/42.9
sequ_4k_1	767	0.891	29.59	366/608	184/341	50.3/56.1
sequ_4k_3	816	1.21	29.69	433/306	151/217	34.9/71.0
sequ_5k_1	718	0.577	27.12	364/445	126/232	34.5/52.2
sequ_5k_2	587	-0.274	29.1	478/499	217/213	45.4/42.6
sequ_5k_3	611	-0.117	31.34	349/494	147/237	42.1/48.0
sequ_6k_1	731	0.657	27.78	433/361	131/252	30.3/69.9
sequ_6k_2	690	0.39	11.83	699/934	183/405	26.1/43.4
sequ_6k_3	603	-0.174	26.51	536/692	192/222	35.9/32.1

Supplementary Table 2: Summary of posterior distributions of all model parameters;, including parameter 'p' as well as Gaussian process components for each site.

Parameter	Estimate	Lower 95% CI	Upper 95% CI
Intercept	-0.571	-0.669	-0.472
site_cwd_zscore	0.850	0.700	0.993
prop_host_count_s	0.679	0.617	0.742
pipo_and_dead_mean_height_s	0.245	0.140	0.351
overall_tpha_s	-0.013	-0.106	0.080
overall_bapha_s	-0.129	-0.294	0.035
site_cwd_zscore : prop_host_count_s	-0.078	-0.184	0.026
site_cwd_zscore : pipo_and_dead_mean_height_s	0.540	0.374	0.702
site_cwd_zscore : overall_tpha_s	-0.190	-0.308	-0.074
site_cwd_zscore : overall_bapha_s	-0.039	-0.227	0.150
prop_host_count_s : overall_tpha_s	0.065	0.014	0.116
prop_host_count_s : pipo_and_dead_mean_height_s	0.014	-0.046	0.072
pipo_and_dead_mean_height_s : overall_bapha_s	-0.081	-0.134	-0.029
site_cwd_zscore : prop_host_count_s : pipo_and_dead_mean_height_s	0.138	0.039	0.236
p	0.019	0.012	0.027
sdgp_gpxysiteeldo_3k_1	2.191	1.468	3.426
sdgp_gpxysiteeldo_3k_2	3.377	2.411	4.852
sdgp_gpxysiteeldo_3k_3	1.966	1.598	2.405
sdgp_gpxysiteeldo_4k_1	2.119	1.714	2.611
sdgp_gpxysiteeldo_4k_2	2.267	1.808	2.818
sdgp_gpxysiteeldo_5k_1	1.463	1.171	1.815
sdgp_gpxysiteeldo_5k_2	1.355	1.089	1.682
sdgp_gpxysiteeldo_5k_3	0.820	0.549	1.153
sdgp_gpxysitesequ_4k_1	1.612	1.323	1.951
sdgp_gpxysitesequ_4k_3	1.468	1.182	1.806

Parameter	Estimate	Lower 95% CI	Upper 95% CI
sdgp__gpxysitesequ__5k__1	1.128	0.902	1.405
sdgp__gpxysitesequ__5k__2	1.452	1.190	1.762
sdgp__gpxysitesequ__5k__3	1.488	1.221	1.802
sdgp__gpxysitesequ__6k__1	1.373	1.102	1.688
sdgp__gpxysitesequ__6k__2	1.604	1.270	2.018
sdgp__gpxysitesequ__6k__3	1.352	1.124	1.607
sdgp__gpxysitesier__3k__1	1.639	1.293	1.976
sdgp__gpxysitesier__3k__2	1.387	1.099	1.716
sdgp__gpxysitesier__3k__3	1.643	1.373	1.962
sdgp__gpxysitesier__4k__1	1.287	1.057	1.550
sdgp__gpxysitesier__4k__2	1.707	1.429	2.020
sdgp__gpxysitesier__4k__3	0.834	0.649	1.041
sdgp__gpxysitesier__5k__1	1.133	0.961	1.331
sdgp__gpxysitesier__5k__2	1.464	1.213	1.751
sdgp__gpxysitesier__5k__3	1.184	0.972	1.429
sdgp__gpxysitestan__3k__1	1.486	1.195	1.823
sdgp__gpxysitestan__3k__2	2.078	1.547	2.650
sdgp__gpxysitestan__3k__3	1.712	1.400	2.082
sdgp__gpxysitestan__4k__1	1.650	1.360	1.969
sdgp__gpxysitestan__4k__2	1.492	1.185	1.875
sdgp__gpxysitestan__5k__1	0.959	0.738	1.220
sdgp__gpxysitestan__5k__2	1.434	1.119	1.790
lscale__gpxysiteeldo__3k__1	122.527	80.974	165.079
lscale__gpxysiteeldo__3k__2	77.212	59.149	95.956
lscale__gpxysiteeldo__3k__3	28.007	21.953	35.176
lscale__gpxysiteeldo__4k__1	20.535	14.849	26.757
lscale__gpxysiteeldo__4k__2	23.312	18.322	29.136
lscale__gpxysiteeldo__5k__1	23.225	17.253	30.002
lscale__gpxysiteeldo__5k__2	27.891	20.834	36.309
lscale__gpxysiteeldo__5k__3	35.535	15.659	68.654
lscale__gpxysitesequ__4k__1	24.365	19.004	30.498

Parameter	Estimate	Lower 95% CI	Upper 95% CI
lscale__gpxysitesequ__4k__3	27.882	22.381	34.025
lscale__gpxysitesequ__5k__1	29.609	21.453	38.143
lscale__gpxysitesequ__5k__2	35.830	28.394	44.061
lscale__gpxysitesequ__5k__3	32.851	23.760	42.647
lscale__gpxysitesequ__6k__1	22.797	16.762	30.881
lscale__gpxysitesequ__6k__2	29.827	23.407	36.888
lscale__gpxysitesequ__6k__3	24.137	18.044	31.738
lscale__gpxysitesier__3k__1	38.782	29.756	77.498
lscale__gpxysitesier__3k__2	20.949	15.023	28.096
lscale__gpxysitesier__3k__3	23.668	19.028	28.425
lscale__gpxysitesier__4k__1	23.326	18.878	27.843
lscale__gpxysitesier__4k__2	24.355	19.638	29.676
lscale__gpxysitesier__4k__3	26.224	18.796	36.376
lscale__gpxysitesier__5k__1	15.239	12.483	18.084
lscale__gpxysitesier__5k__2	22.892	18.612	27.608
lscale__gpxysitesier__5k__3	20.747	16.161	25.277
lscale__gpxysitestan__3k__1	23.627	17.480	30.664
lscale__gpxysitestan__3k__2	29.972	19.919	51.984
lscale__gpxysitestan__3k__3	38.294	31.174	46.354
lscale__gpxysitestan__4k__1	24.316	17.580	32.883
lscale__gpxysitestan__4k__2	26.789	20.752	33.914
lscale__gpxysitestan__5k__1	16.224	11.293	22.050
lscale__gpxysitestan__5k__2	30.206	21.116	45.937

Supplementary Table 3: Reflectance sensitivity of the Micasense Rededge3 camera. The calibration panel value represents the reflectance of the calibration panel for the given wavelength.

Band number	Band name	Center wavelength	Band width	Wavelength range	Panel reflectance
1	blue (b)	475	20	465-485	0.64
2	green (g)	560	20	550-570	0.64
3	red (r)	668	10	663-673	0.64
4	near infrared (nir)	840	40	820-860	0.6
5	red edge (re)	717	10	712-722	0.63

Supplementary Figures

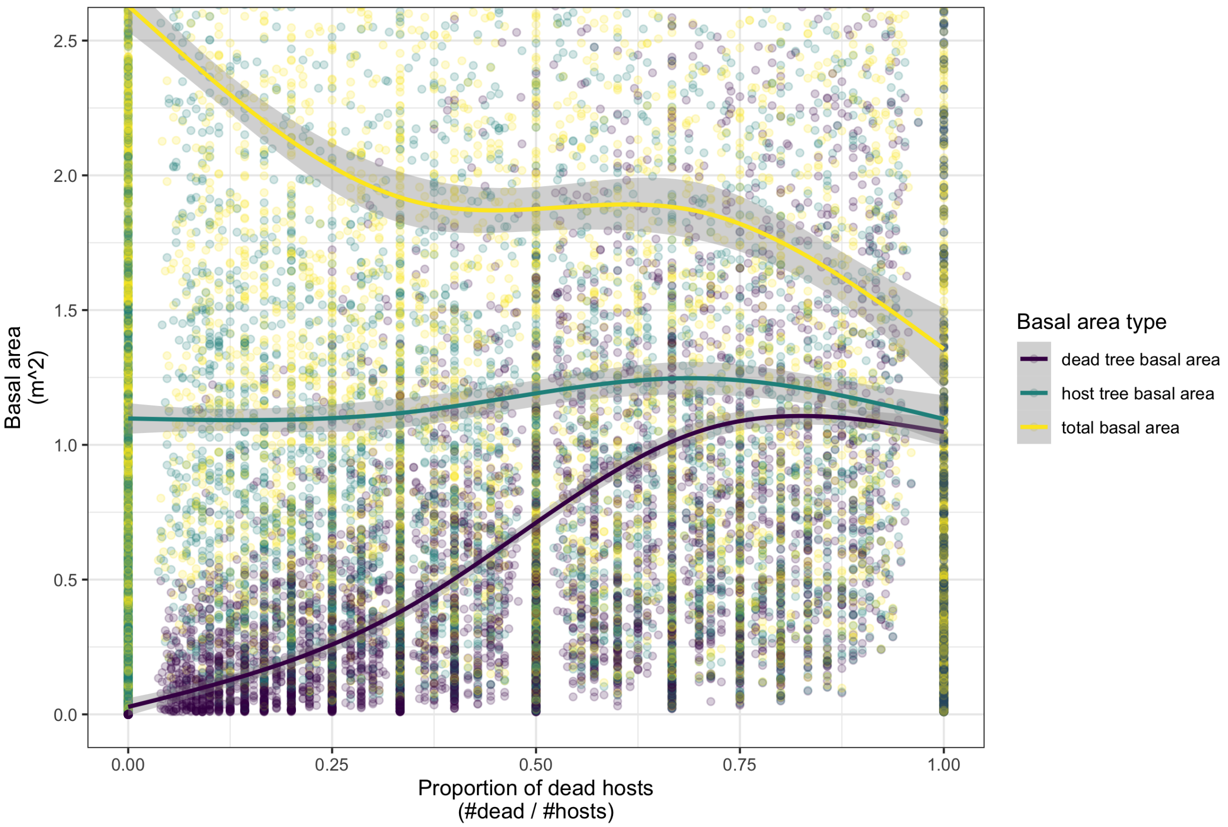

Supplementary Figure 1: The basal area mortality consequences as a function of the proportion of dead host trees (i.e., the response variable of our analysis). Note that the y-axis is constrained in order to highlight the trend in the mean. An increase in the proportion of dead trees (based on count) correlates with an increasing mean quantity of basal area killed. Coupled with the positive effect of mean tree height in a pixel with the probability of host tree mortality, this suggests that larger trees were the actual victim of bark beetle attack, rather than the alternative scenario whereby large trees in a plot act as a siren call that increases the probability of mortality of their smaller neighbors but not themselves.

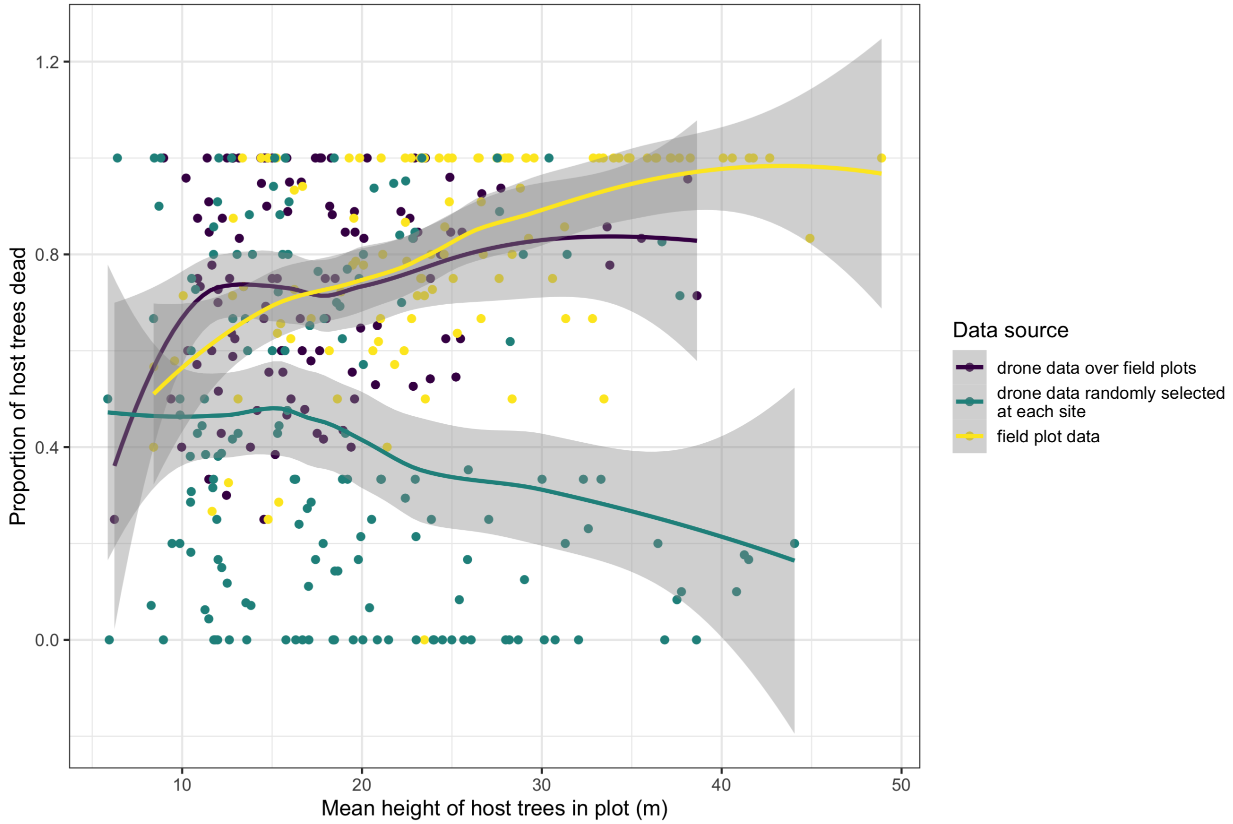

Supplementary Figure 2: The relationship between mean host height and the proportion of host mortality based on the data source. Field plot data come from 1, drone data over field plots comes from this study, but with trees subsetted to only those identified within the ground footprints of the field plots of 1, and drone data randomly selected at each site come from this study but are randomly selected 20 x 20 m cells (5 from each site) from the Level 4 product. The tree height/mortality relationship appears similar for trees in the coincident field plots (both field-derived and drone-derived tree measurements), increasing confidence in the drone-derived forest measurements. The drone data representing the broader context around the coincident field plots show a dramatically different relationship, highlighting the benefit of extending the field surveys with aerial drone surveys.

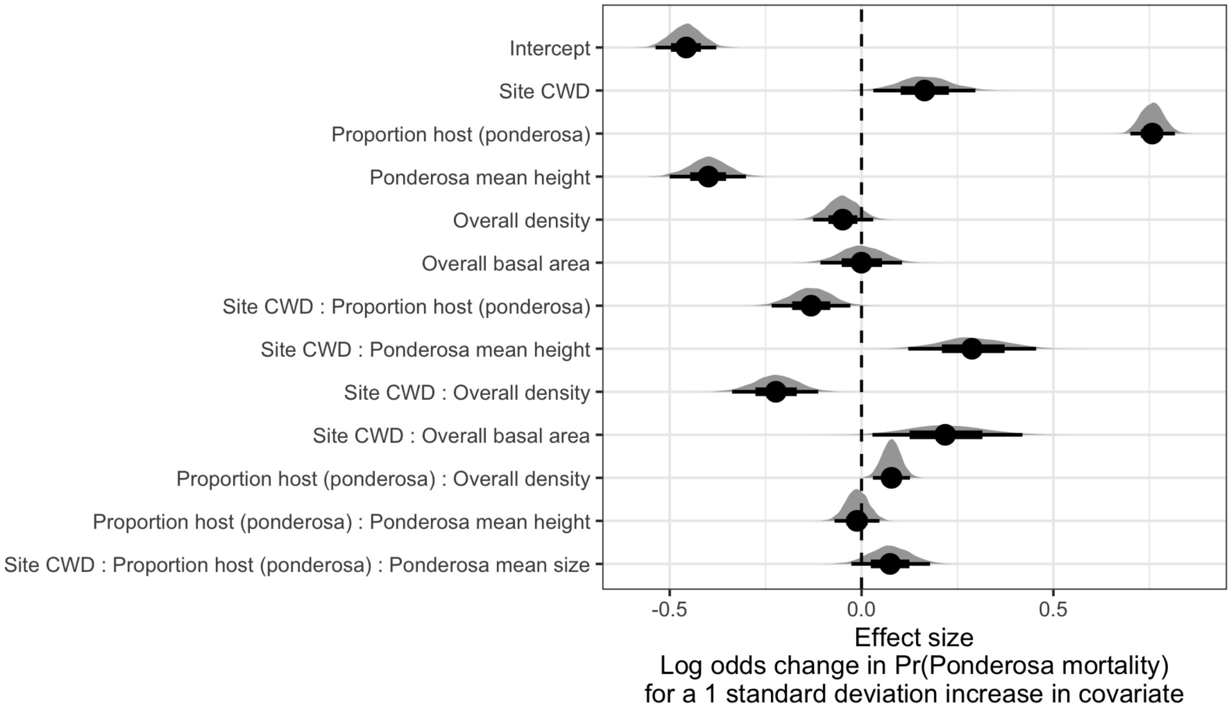

Supplementary Figure 3: Using uncalibrated tree heights, the posterior distributions of effect size from zero-inflated binomial model predicting the probability of ponderosa pine mortality in a 20 x 20-m cell given forest structure characteristics and site-level climatic water deficit (CWD). The gray filled area for each model covariate represents the probability density of the posterior distribution, the point underneath each density curve represents the median of the estimate, the bold interval surrounding the point estimate represents the 66% credible interval, and the thin interval surrounding the point estimate represents the 95% credible interval.

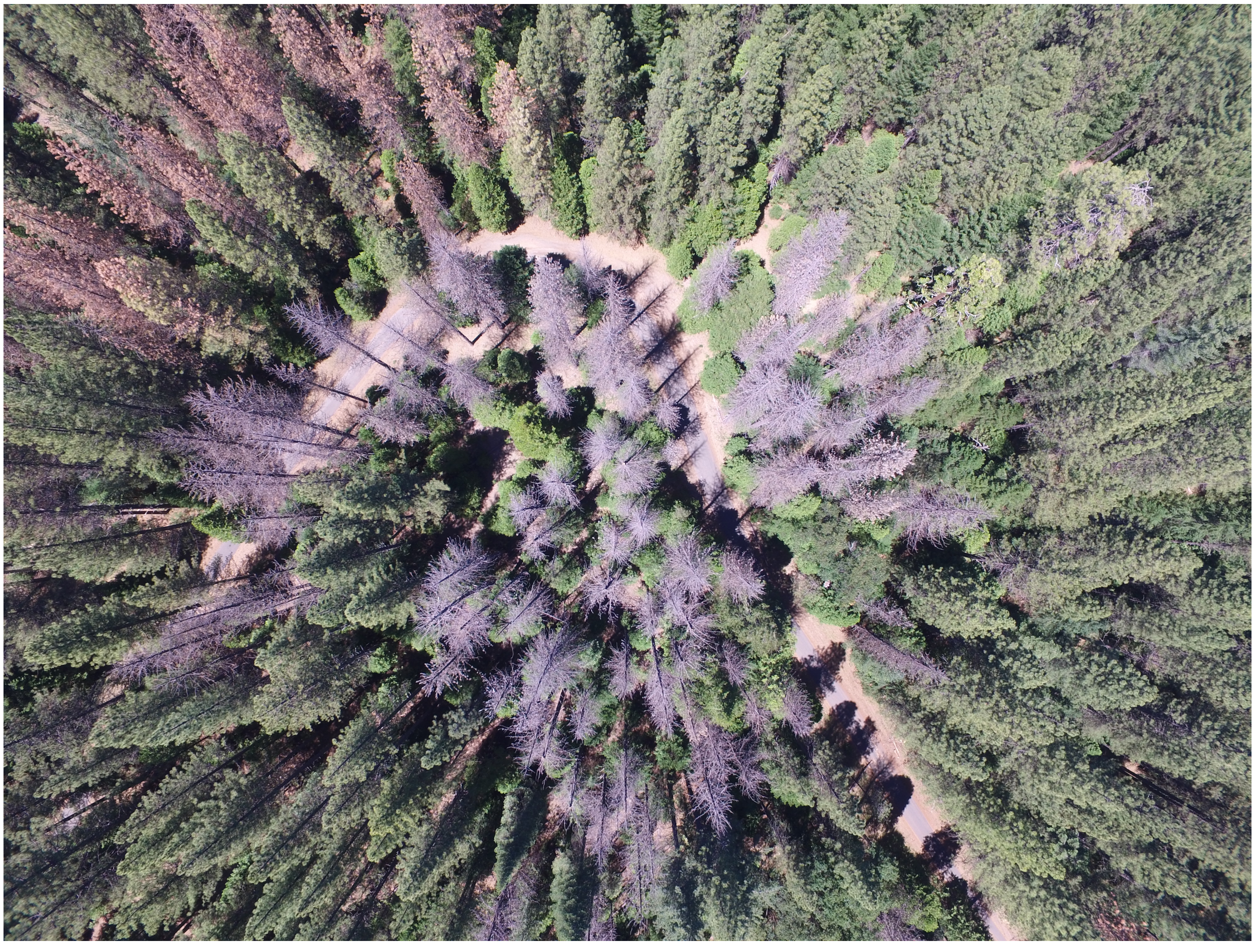

Supplementary Figure 4: An example of a raw red/blue/green (RGB) image capture from the DJI Zenmuse X3 camera.

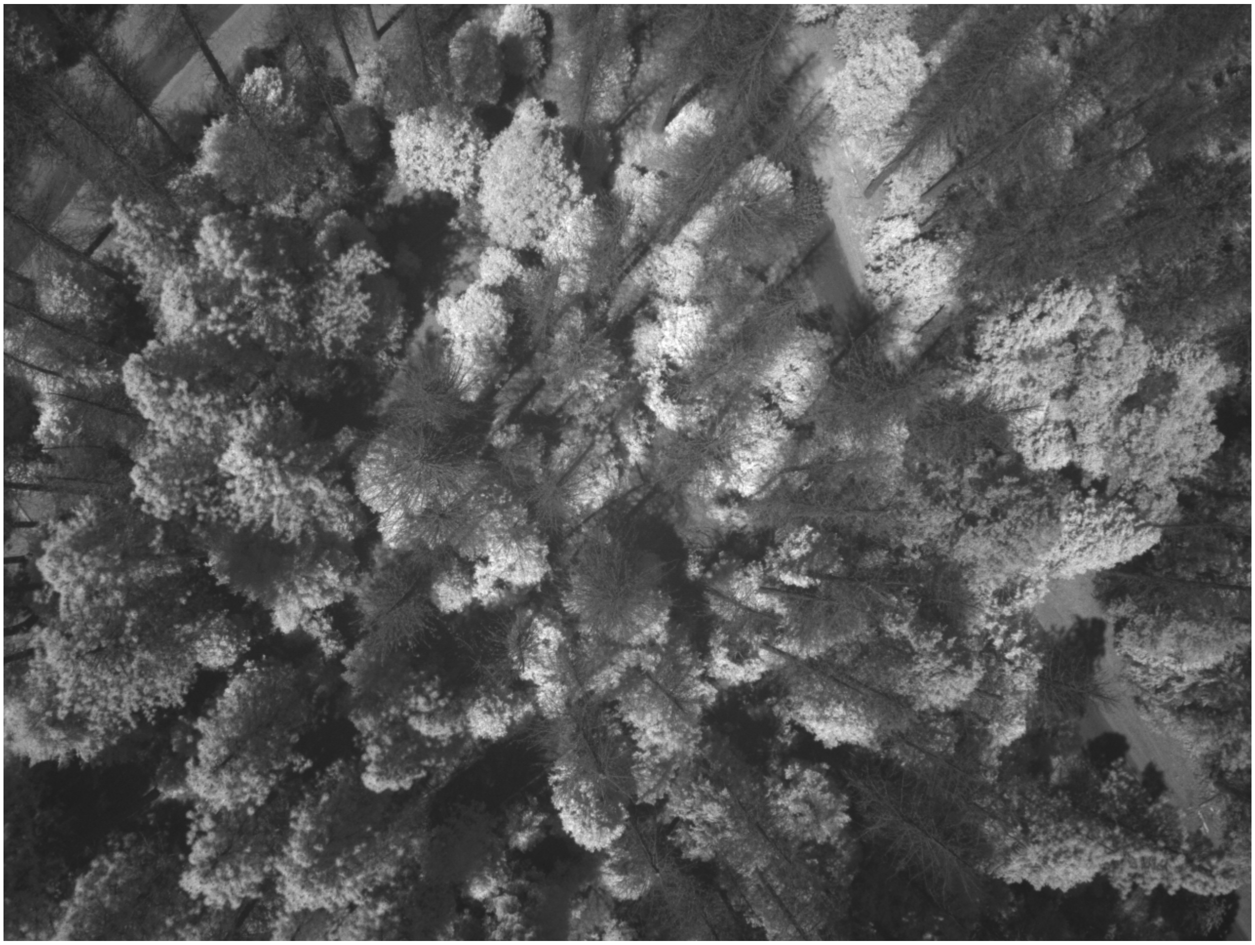

Supplementary Figure 5: An example of a raw image capture in the near infrared band from the Micasense Rededge camera.

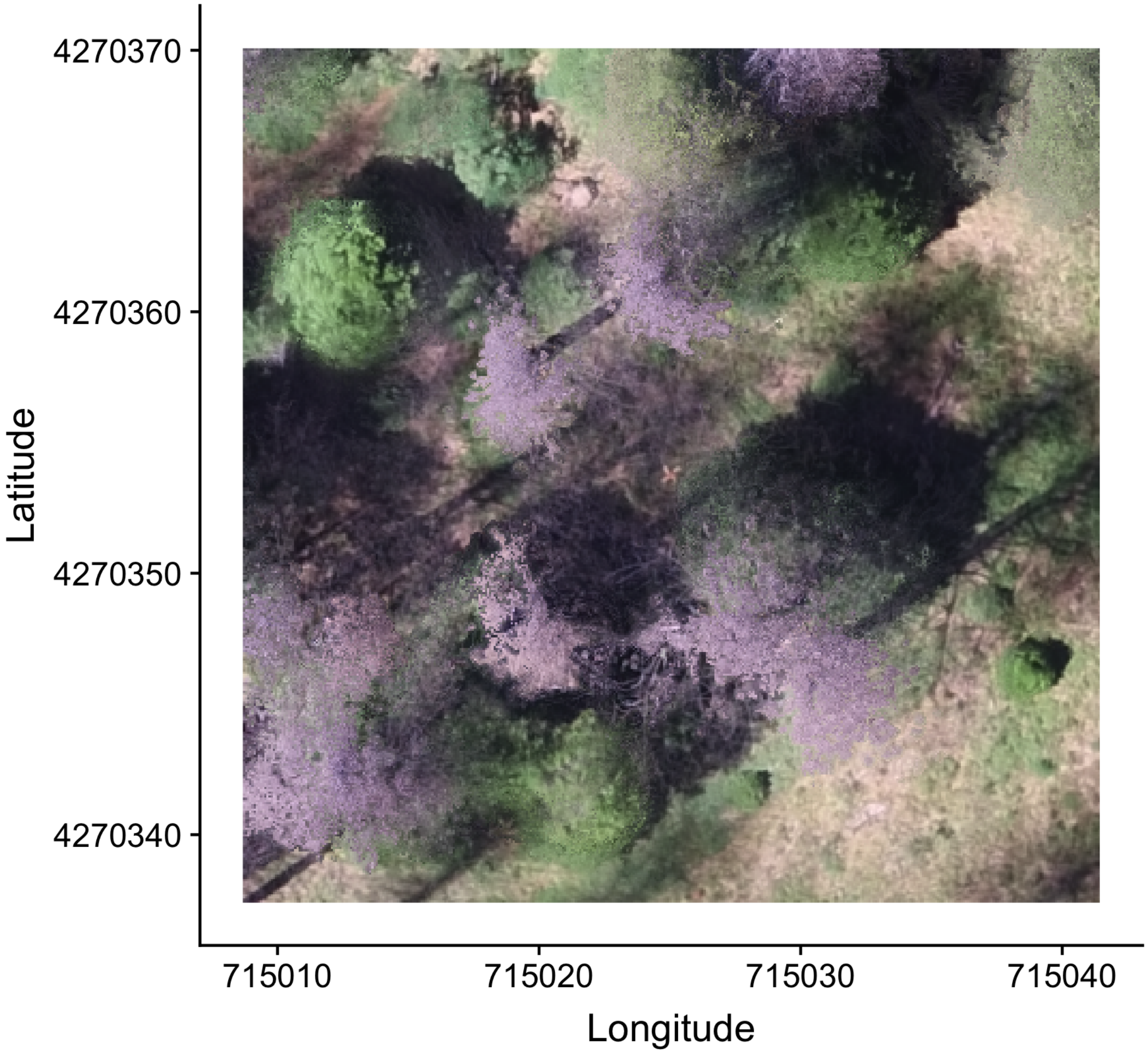

Supplementary Figure 6: A zoomed-in version of the Level 1 orthomosaic depicting a plot center marked with an orange X made out of two strips of drop cloths (approximately 100 x 15 cm per strip). The fabric was laid over the rebar monuments marking each of the 5 plot centers at each of the 32 sites.

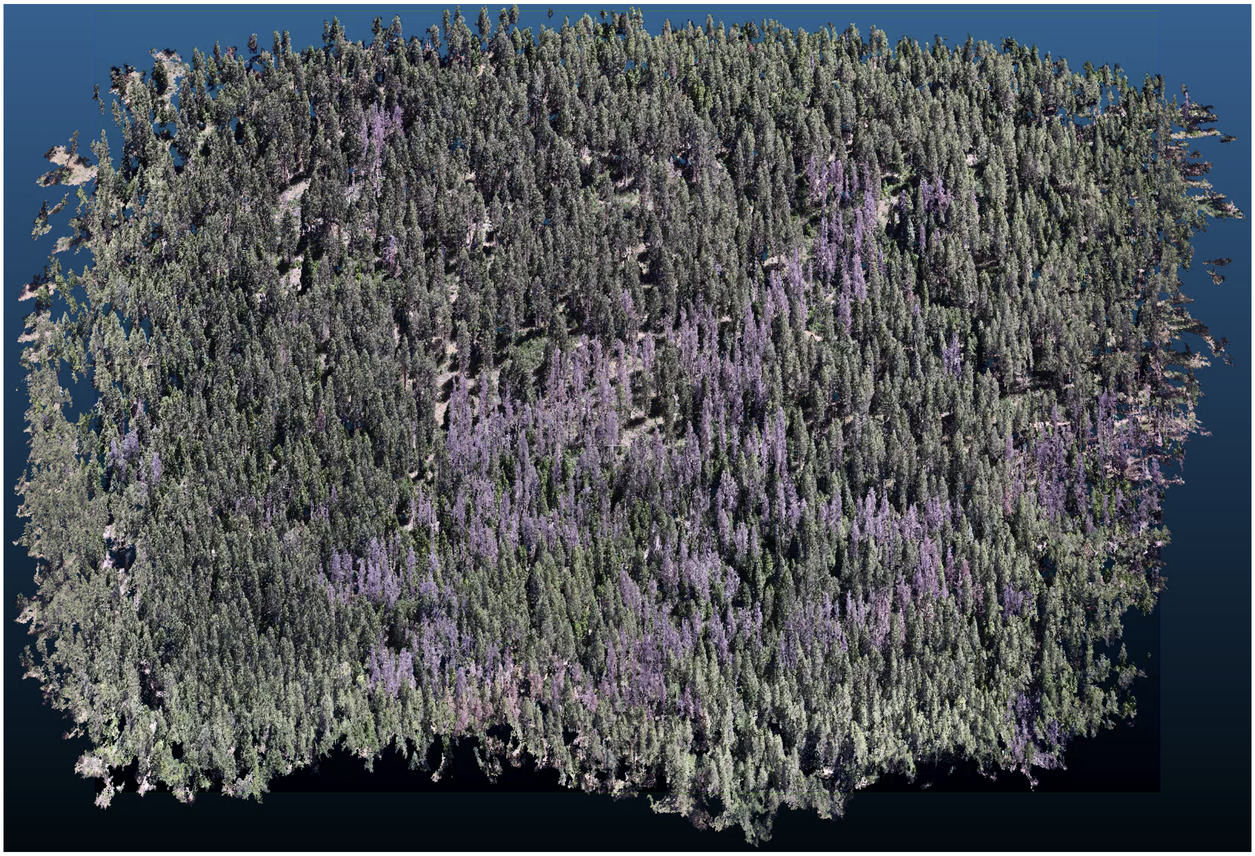

Supplementary Figure 7: A dense point cloud representing ~40 hectares of forest is generated using Structure from Motion (SfM) processing of ~1900 images. The dense point cloud z- position represents the ground elevation plus the vegetation height.

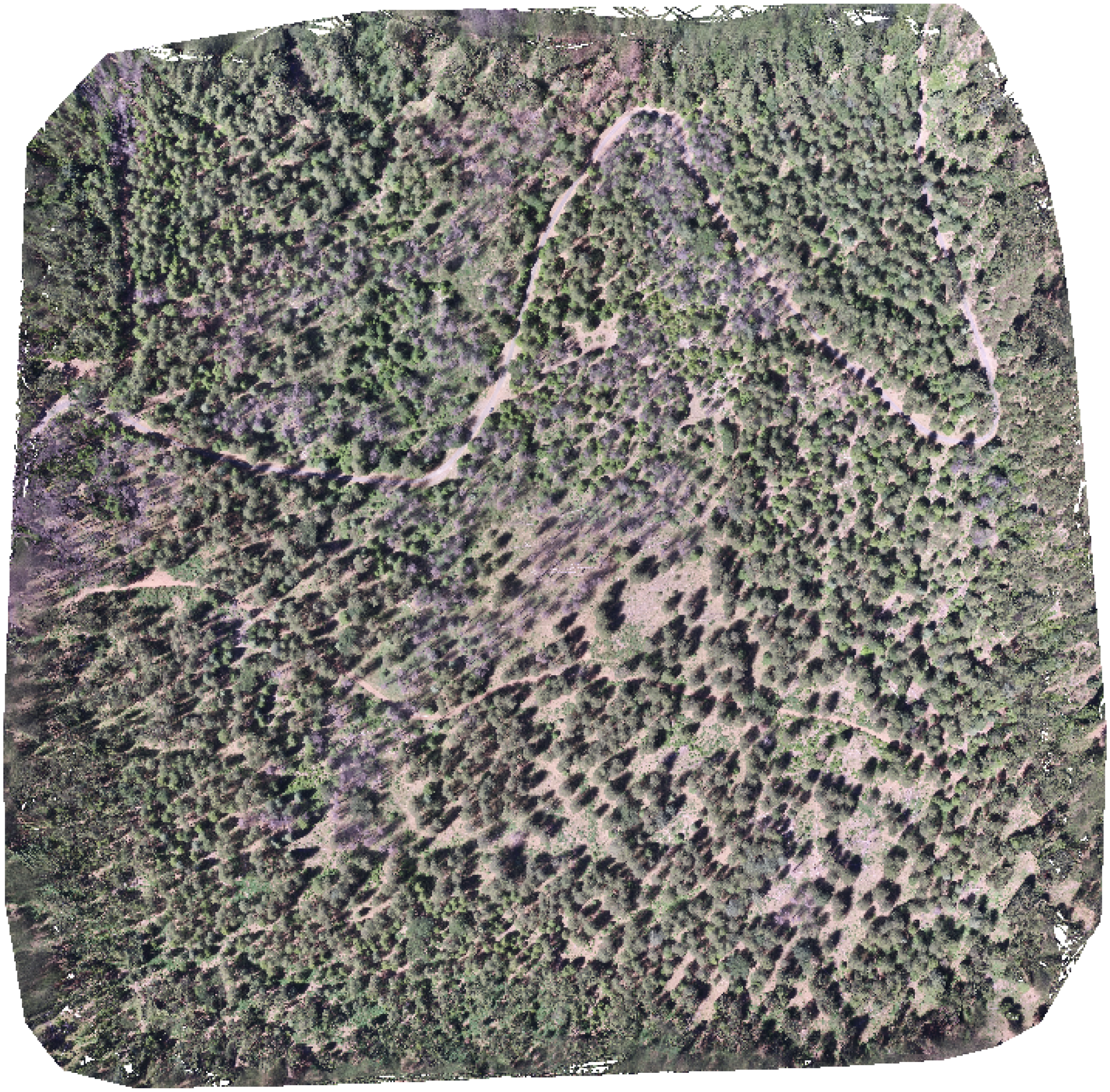

Supplementary Figure 8: The orthomosaic for each of the 32 sites is generated with the Structure from Motion (SfM) processing, showing a top-down view of the whole survey area such that distances between objects in the scene are preserved and can be measured. Depicted is an example red-green-blue orthomosaic for one of the 32 sites covering approximately 40 hectares. The resolution of the original orthophoto is approximately 8cm per pixel, and has been considerably coarsened in this depiction.

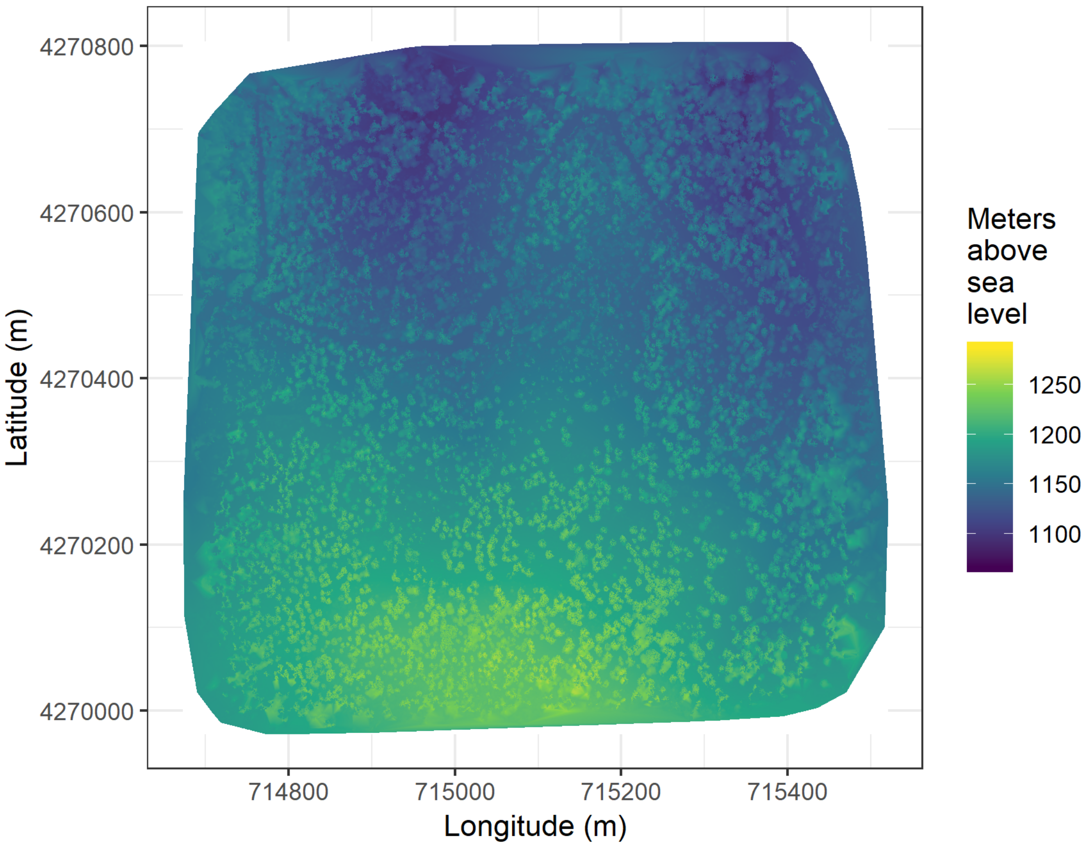

Supplementary Figure 9: The digital surface model (DSM) is a 2-dimensional representation of the dense point cloud generated using structure from motion (SfM) processing. The DSM represents the ground elevation plus the vegetation height.

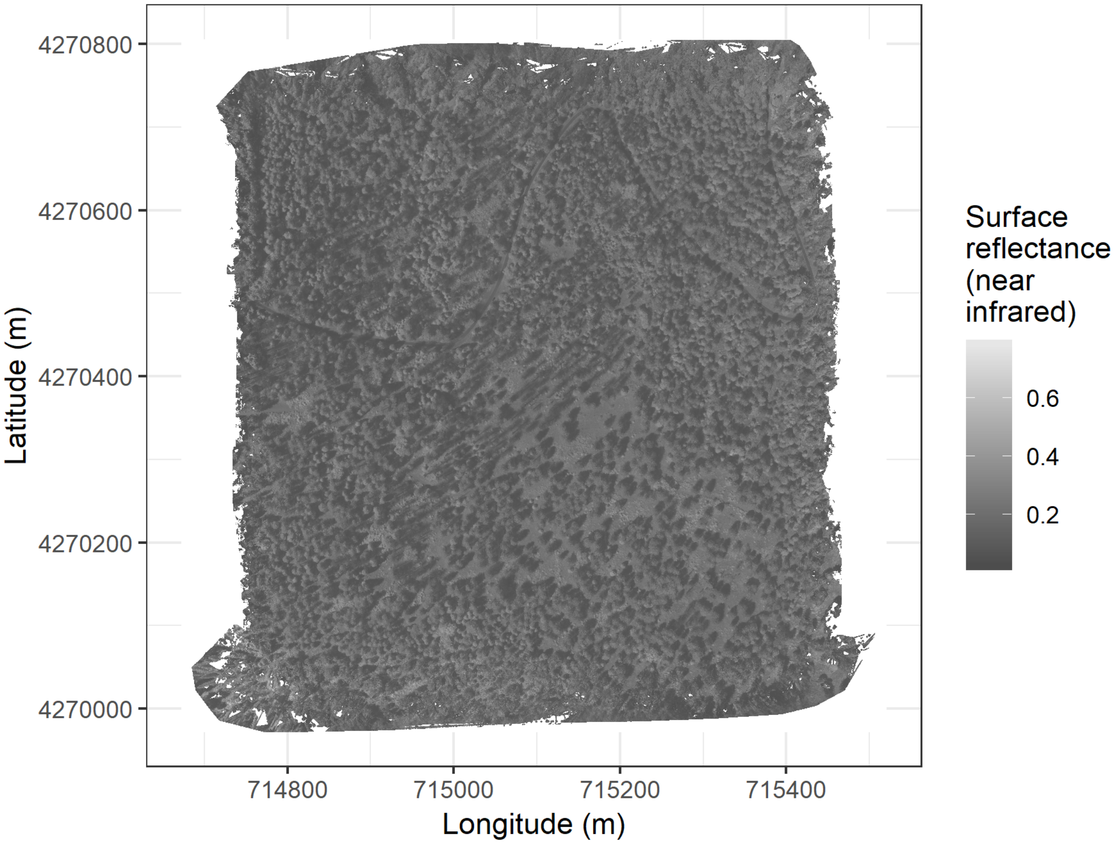

Supplementary Figure 10: A radiometrically-corrected orthomosiac of ‘near infrared’ surface reflectance originally captured by the Micasense Rededge camera with corrections performed during the SfM photogrammetry processing using Pix4DMapper.

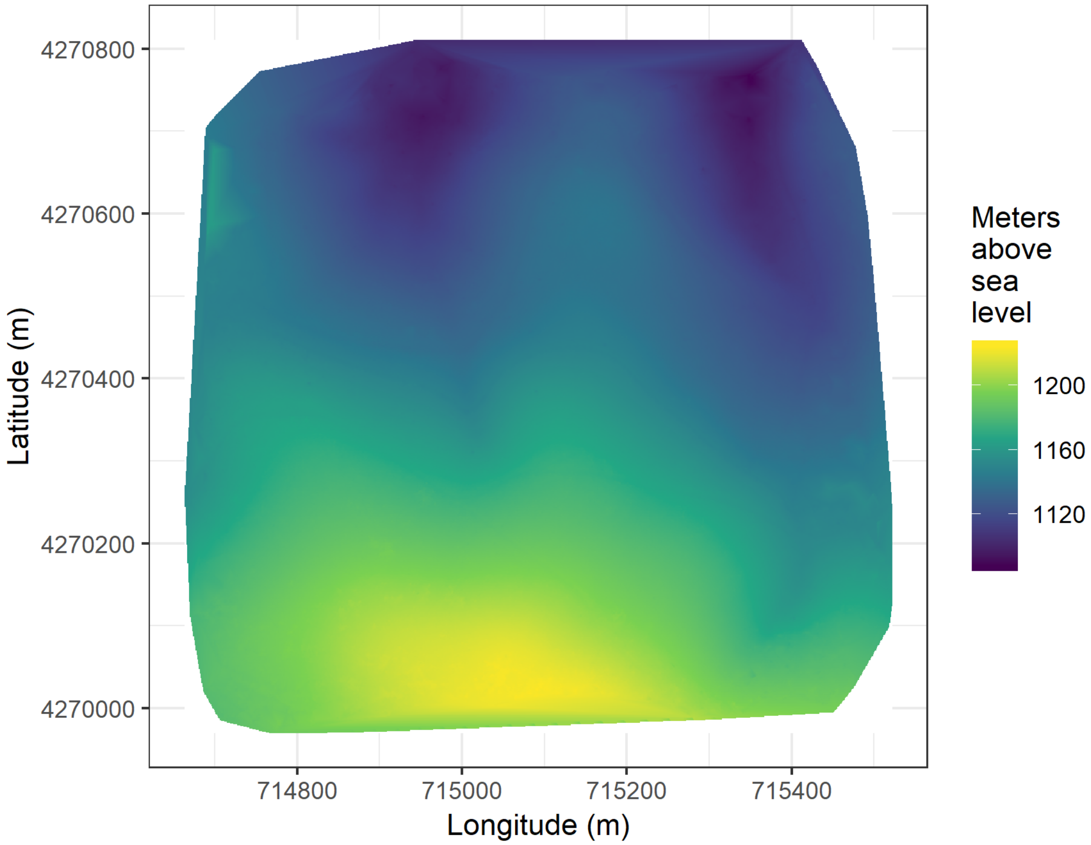

Supplementary Figure 11: The digital terrain model (DTM) is generated by processing the dense point cloud using the cloth simulation filter algorithm,[2] which classifies points as ‘ground’ or ‘not-ground’ and then interpolates the ‘ground’ elevation using Delaunay triangulation for the rest of the dense point cloud footprint. The DTM represents the ground elevation without any vegetation.

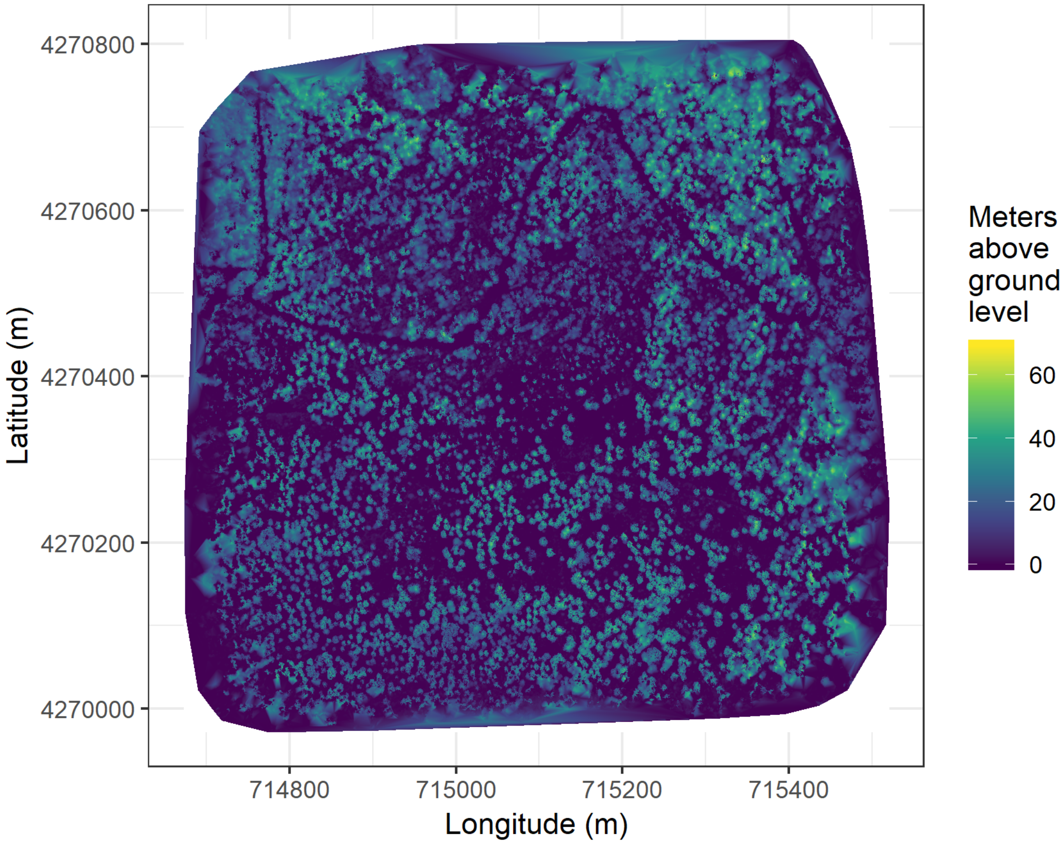

Supplementary Figure 12: The canopy height model (CHM) is generated by subtracting the digital terrain model from the digital surface model. The CHM represents the height of all of the elevation above ground level.

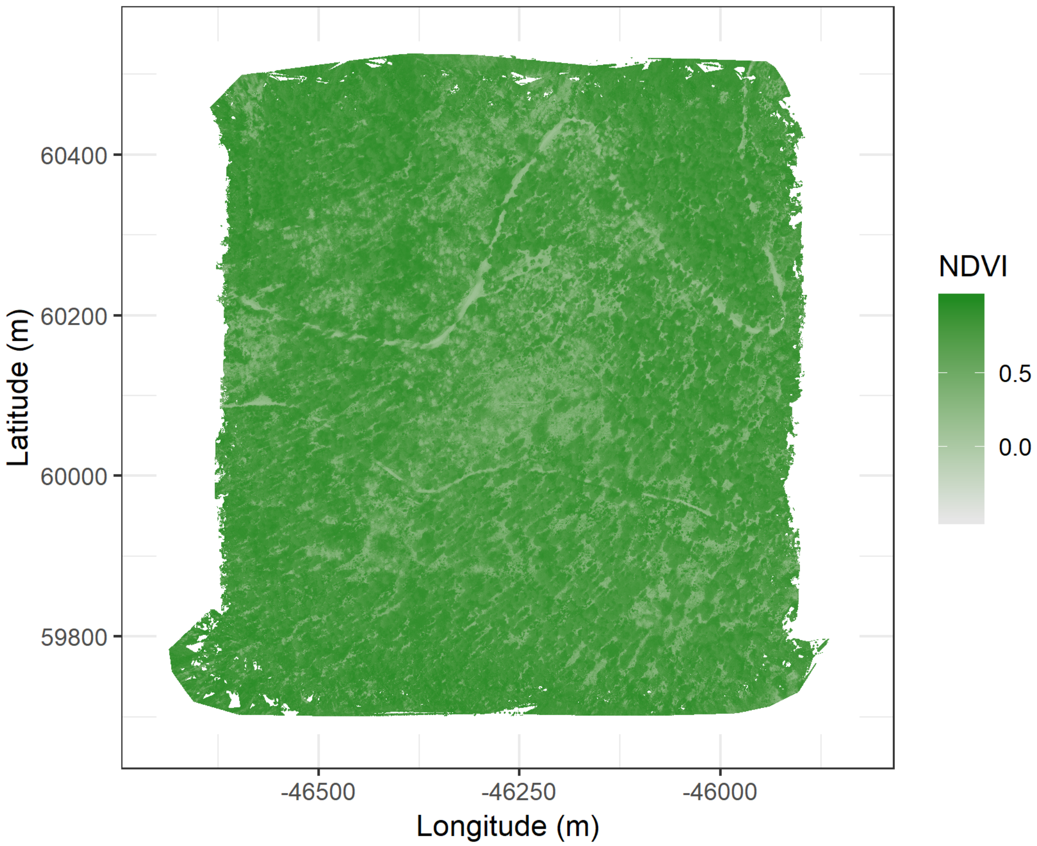

Supplementary Figure 13: An example map of Normalized Difference Vegetation Index[3] derived by combining the Level 2 radiometrically-corrected ‘red’ image and the Level 2 radiometrically-corrected ‘near infrared’ image.

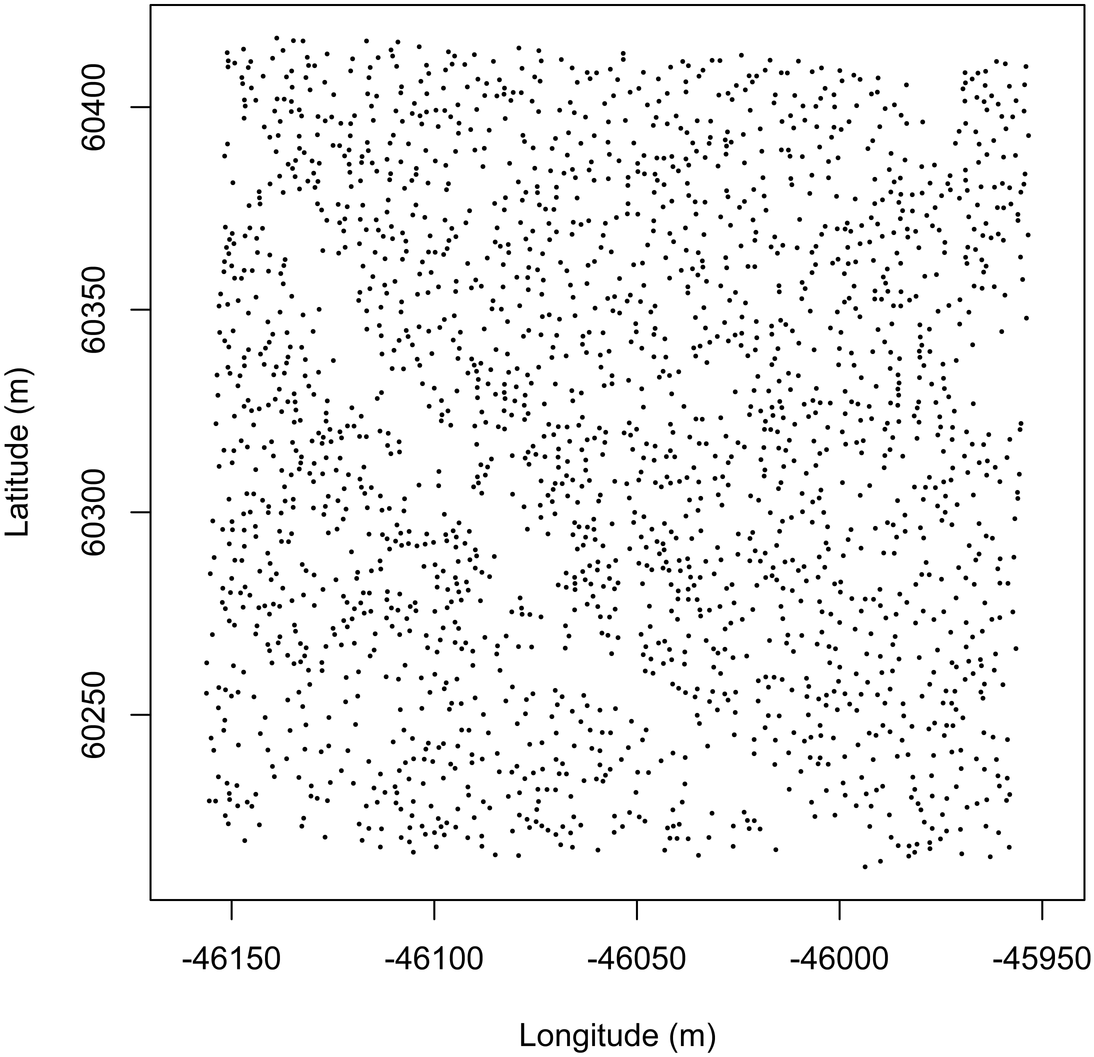

Supplementary Figure 14: Tree locations are detected using the `lmfx`[4] treetop detection algorithm on the dense point cloud.

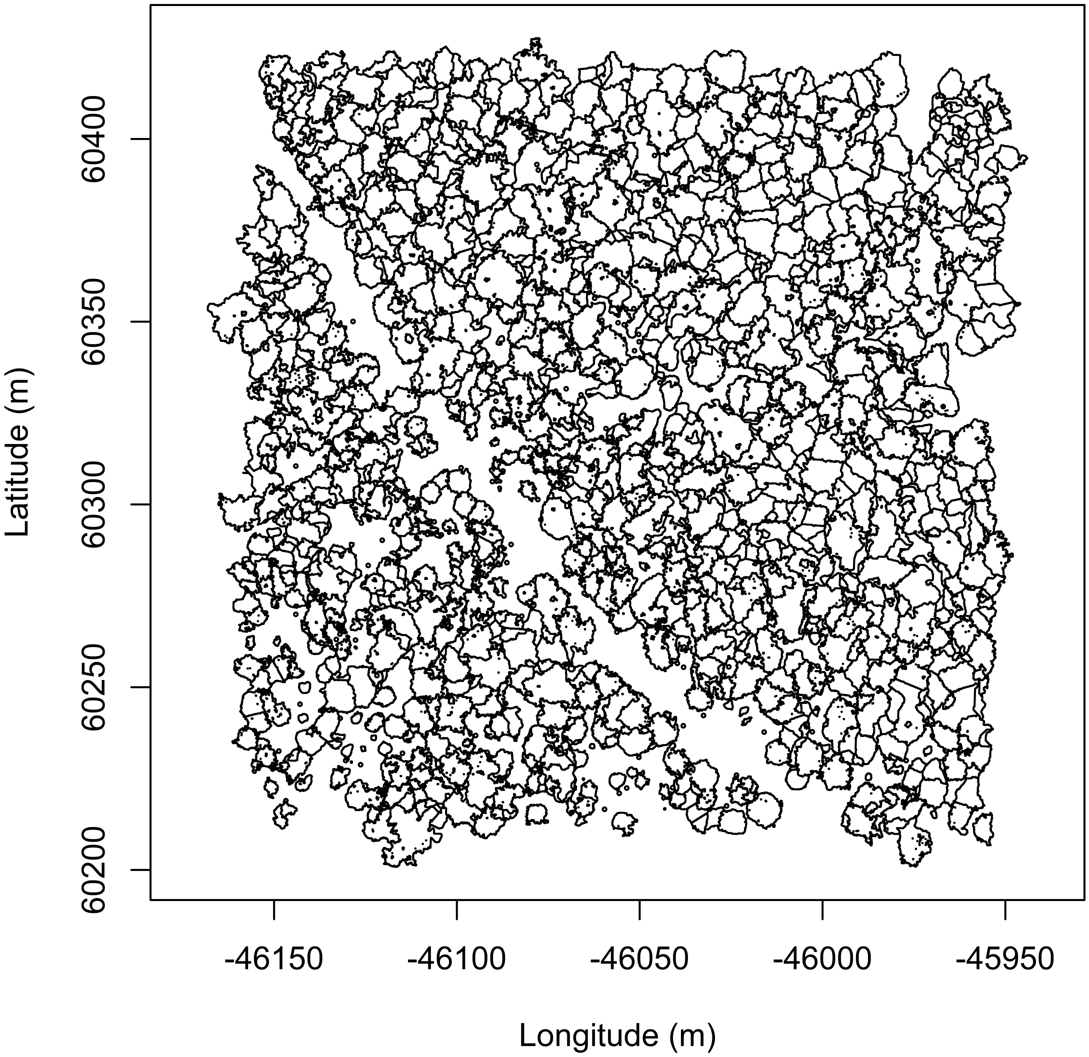

Supplementary Figure 15: Individual crowns are delineated using a marker controlled watershed segmentation algorithm[5,6] on the canopy height model (CHM) using the detected tree locations as a priority map. If the algorithm failed to delineate a crown for a tree that was identified in the tree detection step, a circular crown with a 0.5m buffer centered on point location of the detected tree was added as a crown.

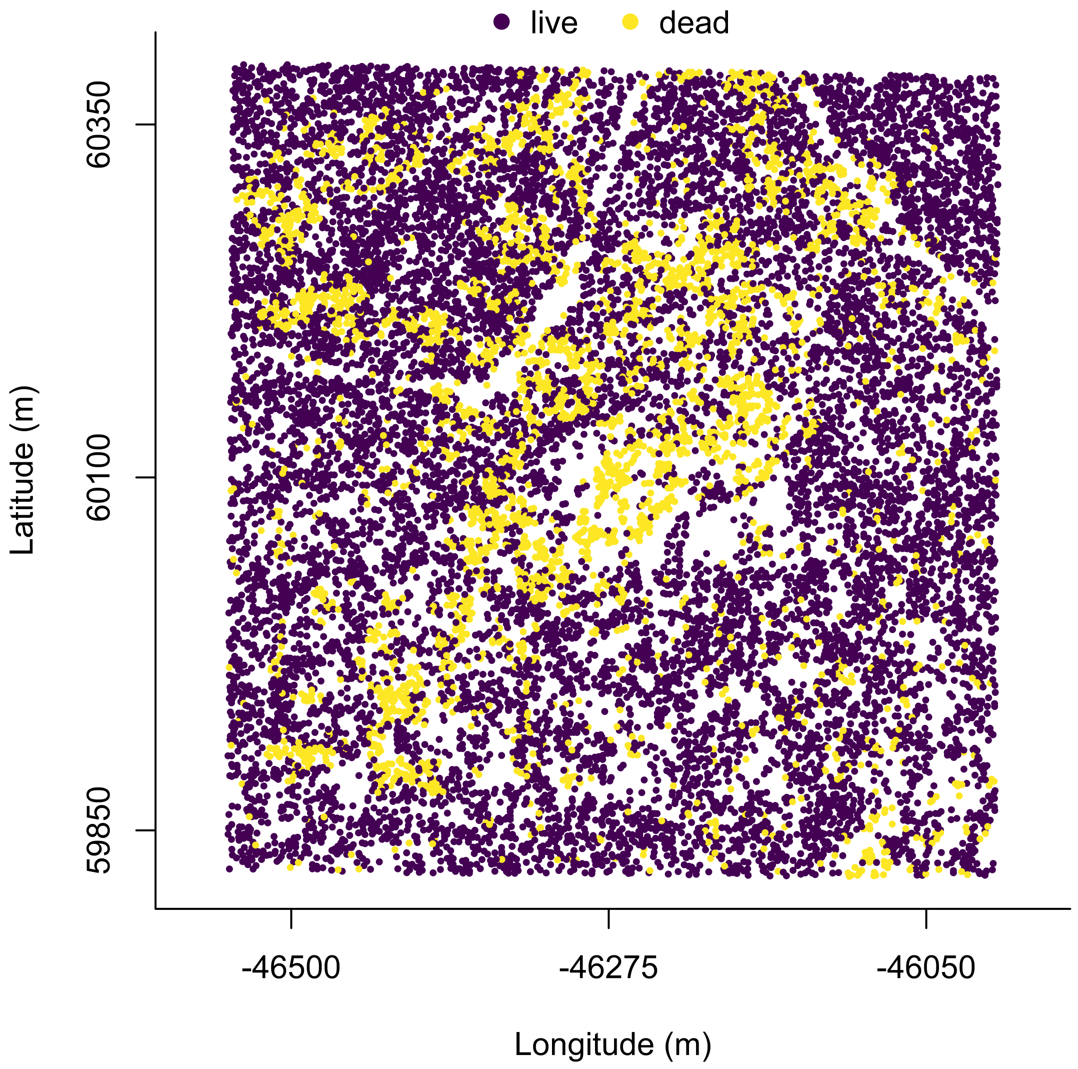

Supplementary Figure 16: Each tree is classified as live or dead by extracting the pixel values from the 5 narrow bands of the Rededge3 camera (and 5 derived bands– see methods) in the orthomosaic within each segmented tree crown of the detected trees, taking their mean value, and using those means to predict live/dead status with a boosted logistic regression previously trained on a hand-classified set of segmented crowns from across the study area.

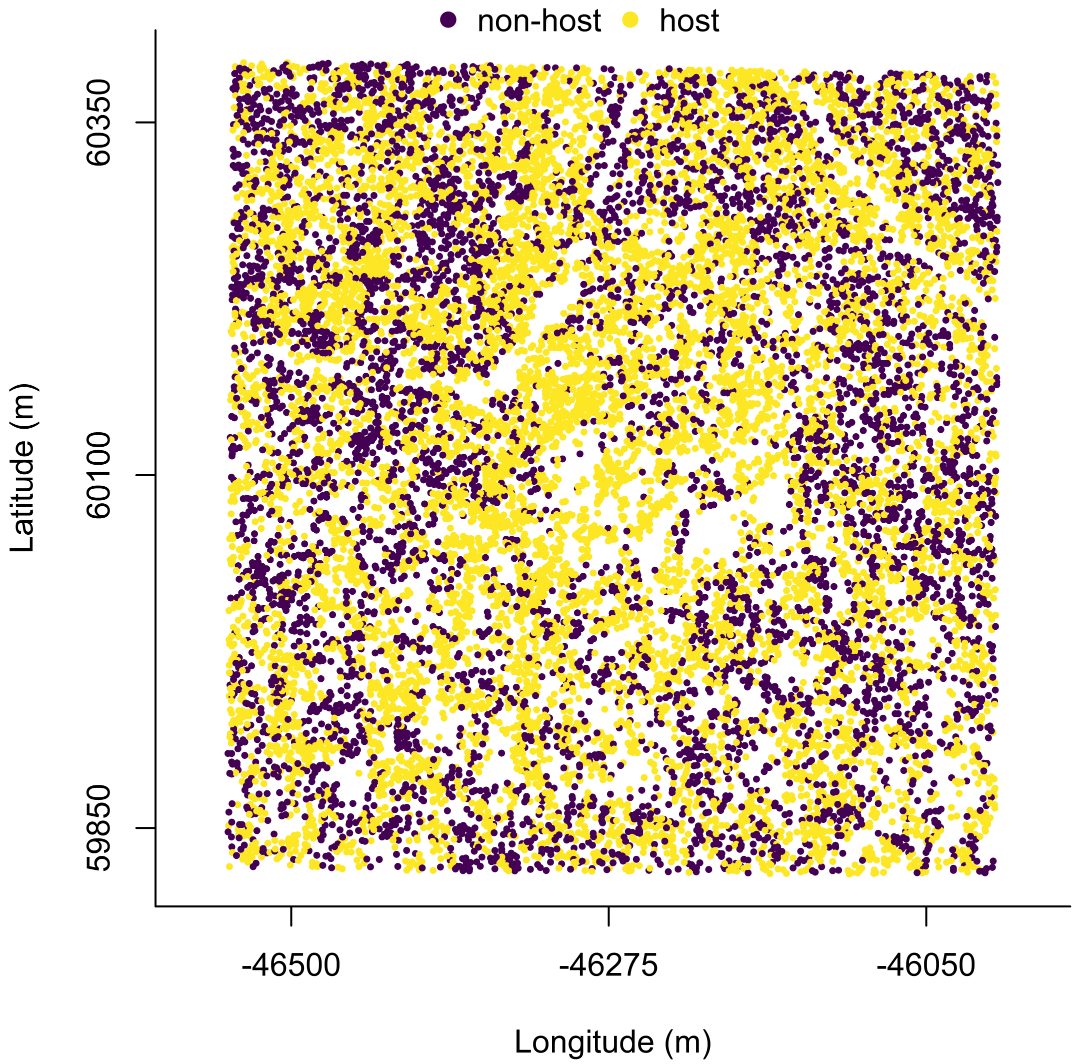

Supplementary Figure 17: For each live tree, we classified its species using the same means of extracted pixel values across the 5 Rededge3 narrow bands (and 5 derived bands) as predictors in a regularized discriminant analysis previously trained on a hand-classified set of segmented crowns from across the study area.

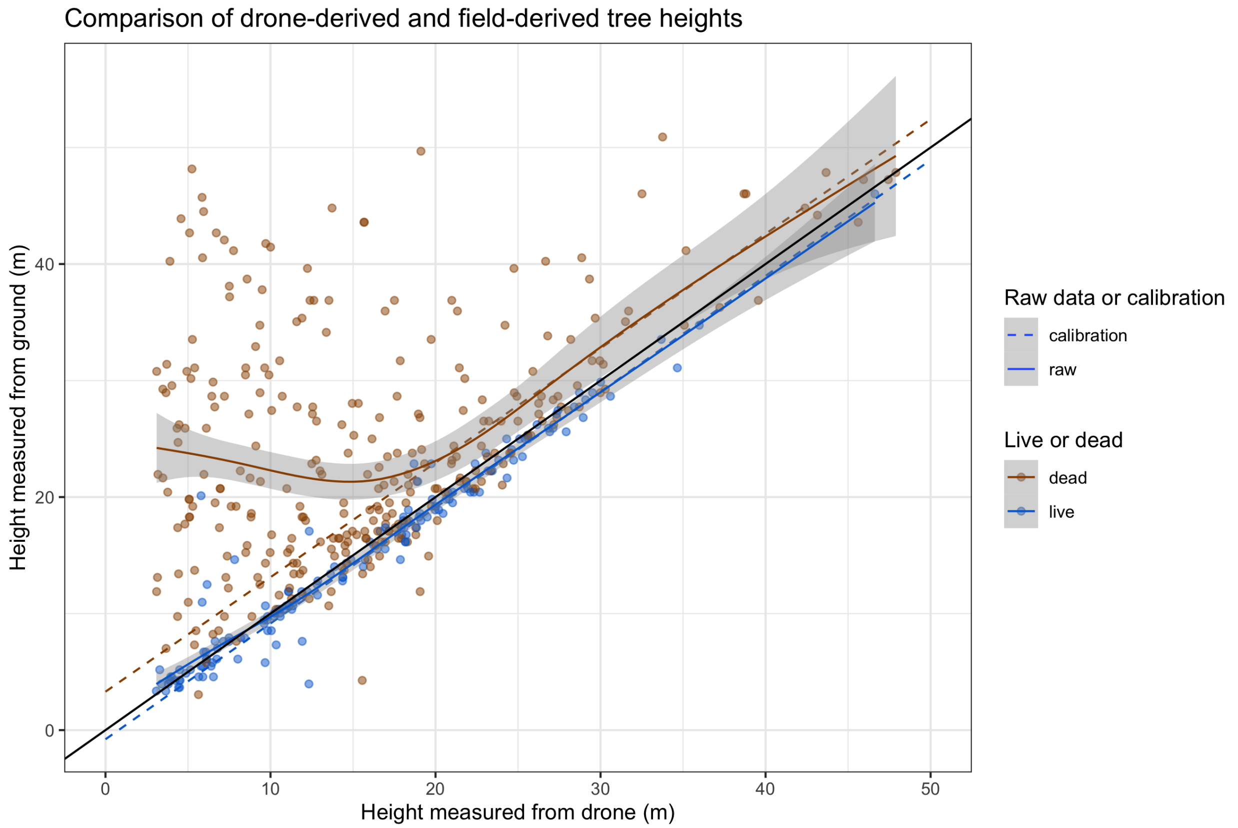

Supplementary Figure 18: We identified 451 field-measured trees in the Level 3b drone-derived data representing segmented and classified trees. Comparing the field- versus drone-measured tree heights for the live and dead trees, we see good agreement for live trees (albeit a small, consistent overestimate of height), and a somewhat good agreement for trees for which the drone-measured height was greater than 20 m (albeit a small, consistent underestimate of height). The solid lines and confidence ribbons represent a General Additive Model fit to these data. Using a linear relationship for trees for which the drone-measured height was greater than 20 m, we modeled the difference between field- and drone-measured height as a function of drone-measured height and extended this relationship to all drone-measured heights (the dashed line). This represents a conservative correction of the drone-measured tree heights.

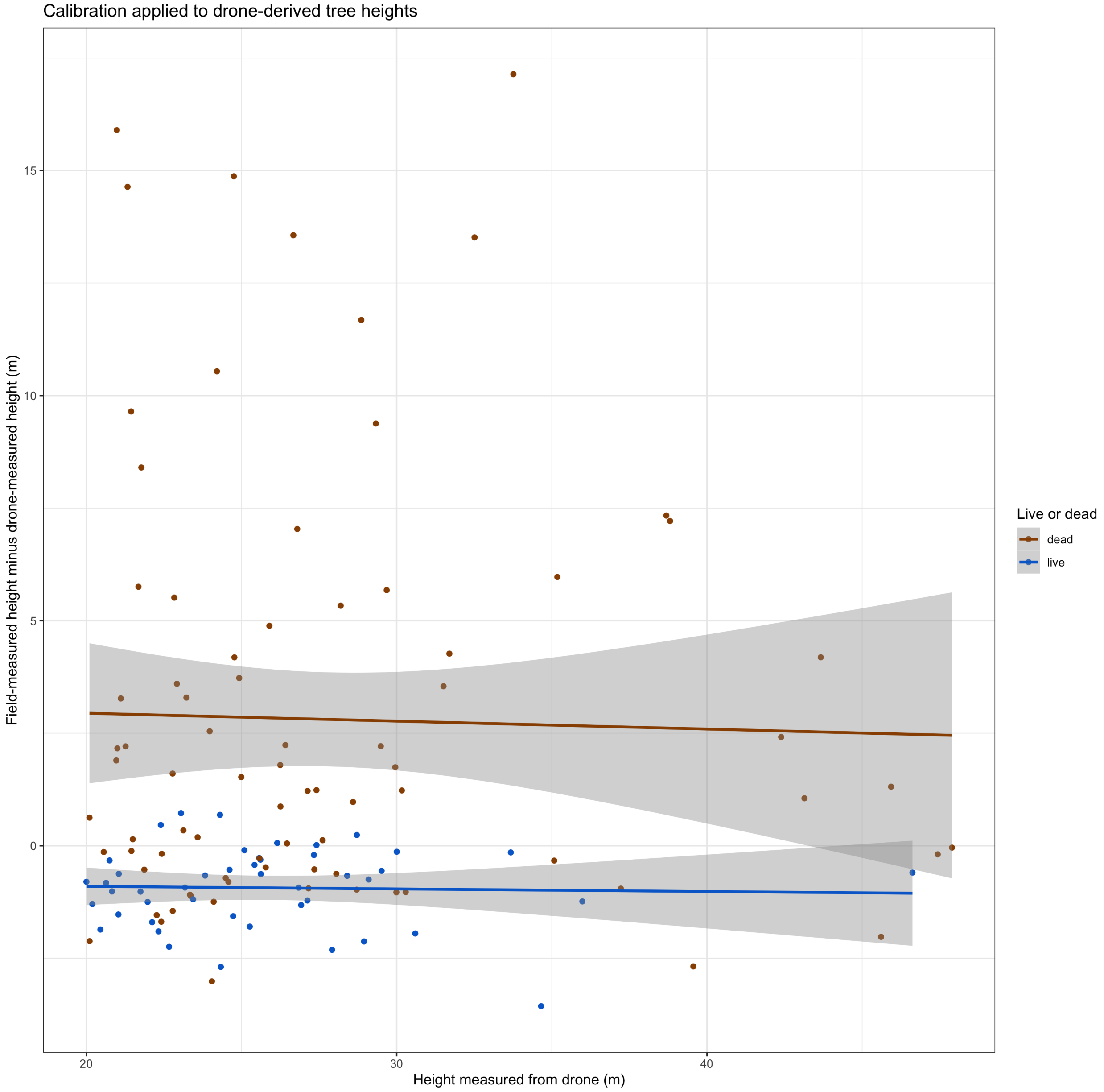

Supplementary Figure 19: We applied a conservative correction to live and dead tree heights in our dataset based on the relationship between the difference between field-measured and drone-measured tree heights as a function of drone-measured tree height. Heights of trees classified as 'dead' were augmented slightly, while heights of trees classified as 'live' were reduced slightly.

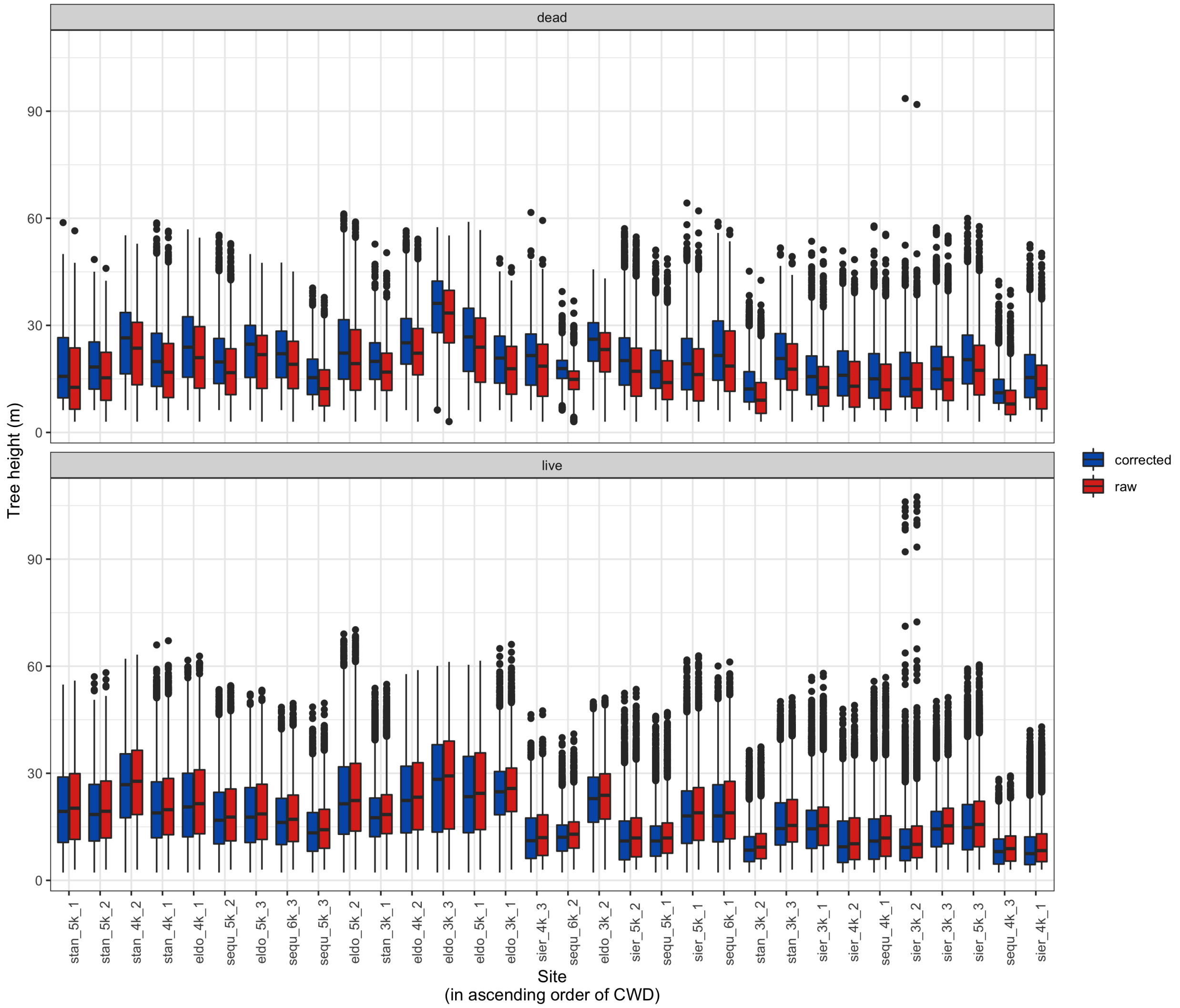

Supplementary Figure 20: Original height distributions for individual classified trees in each site, and the height distributions after the Level 3B calibration. Effect on dead trees .

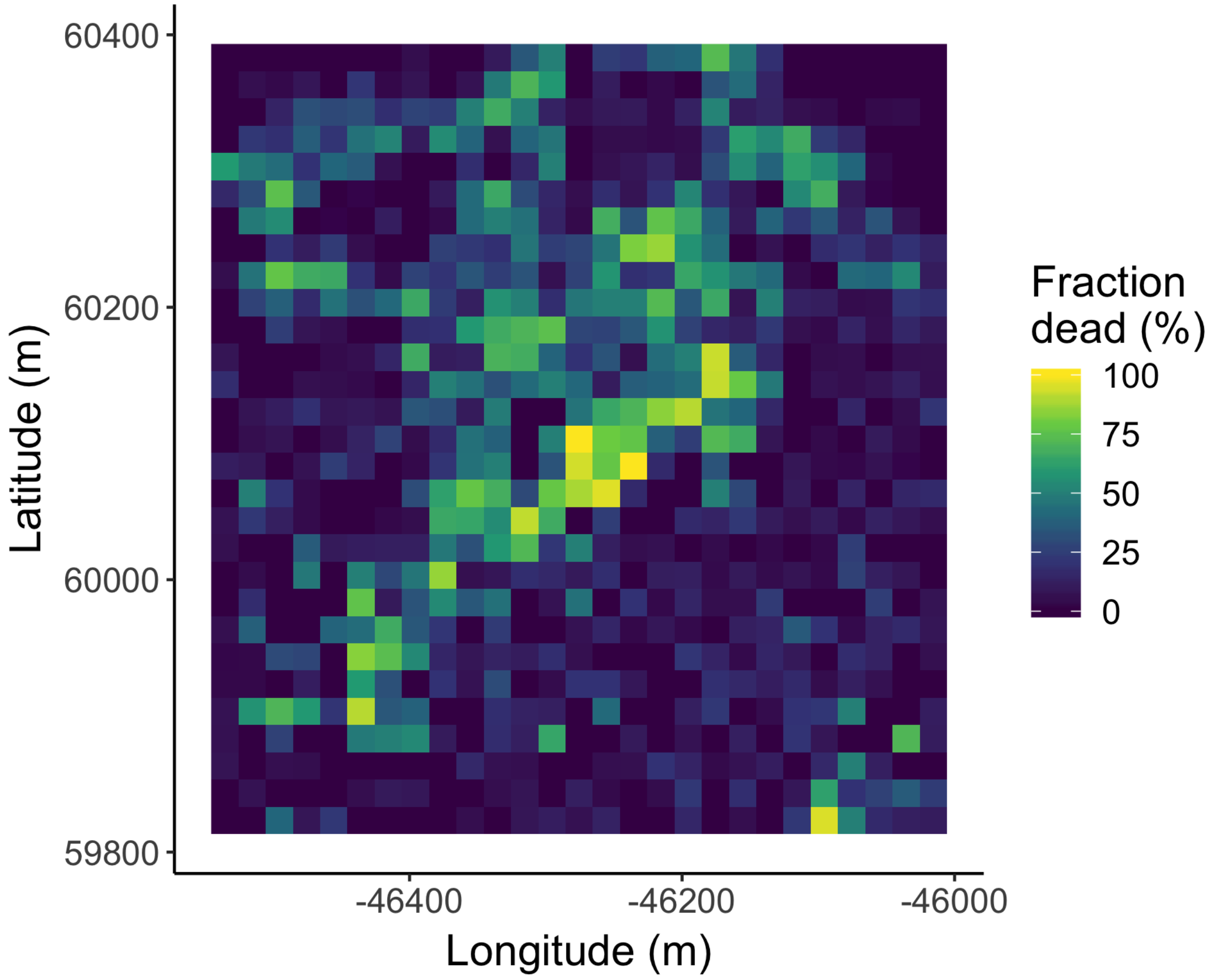

Supplementary Figure 21: We rasterized the individual tree data by aggregating values to 20 x 20 m cells. This example shows the proportion of dead trees per cell for the same example site as in the previous figures.

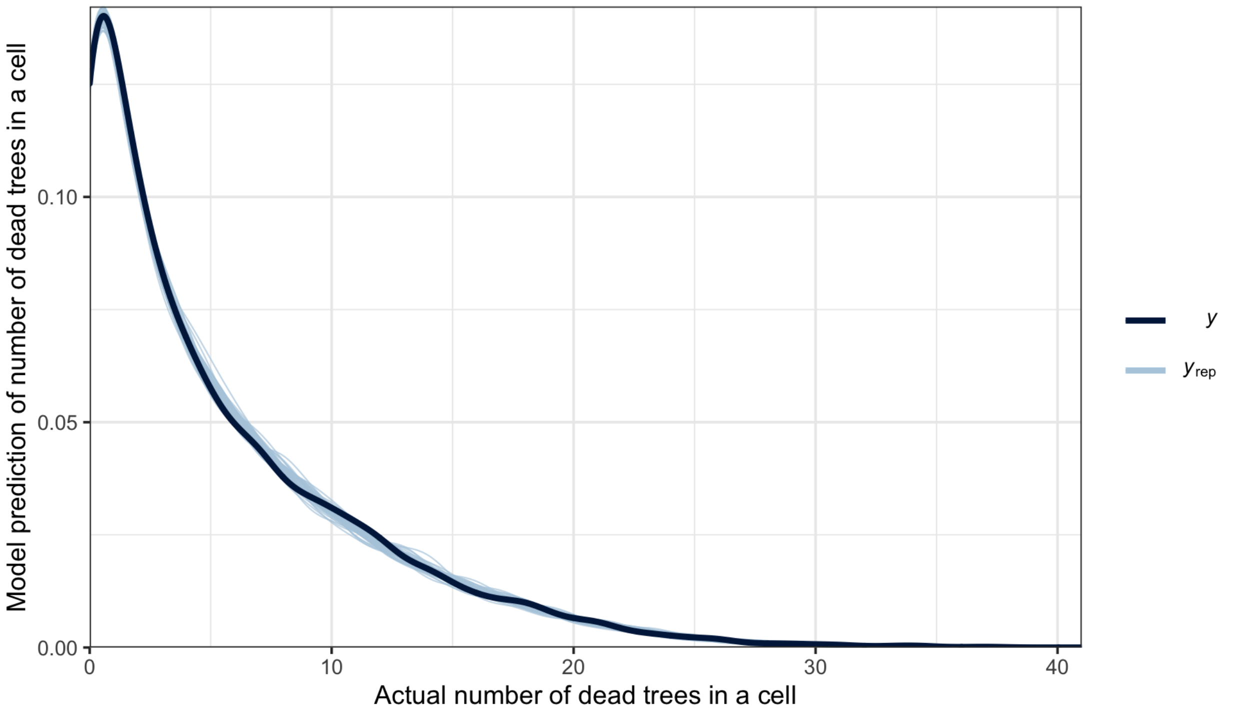

Supplementary Figure 22: Posterior predictive checks of the model presented in Eq. 1 (main text) showing good agreement between the actual number of dead trees in the 20 x 20 m cells and 50 posterior predictions from the model.

Supplementary Methods

Instrumentation

Imagery was captured using a DJI Zenmuse X3 RGB camera[7] and a Micasense RedEdge3 5-band multispectral camera.[8] We mounted both of these instruments simultaneously on a DJI Matrice 100 aircraft[9] using the DJI 3-axis stabilized gimbal for the Zenmuse X3 camera and a Micasense angled fixed mount for the RedEdge3 camera. The gimbal and the angled fixed mount ensured both instruments were nadir-facing during image capture. Just prior to or after image capture at each site, we calibrated the RedEdge3 camera by taking an image of a calibration panel on the ground in full sun with known reflectance values for each of the 5 narrow bands (Supplementary Table 3).

Flight protocol

Image capture was conducted as close to solar noon as possible to minimize shadow effects (varying primarily due to site accessibility; always within 4 hours, usually within 2 hours). Prior to the aerial survey, two strips of bright orange drop cloth (~100cm x 15cm) were positioned as an “X” over the permanent monuments marking the center of the 5 field plots from 1.

For each of the 32 sites (containing 5 plots each), we captured imagery over the surrounding ~40 hectares of forested area using north-south aerial transects. For three sites, we surveyed less surrounding area in order to maintain visual and radio communication with the aircraft during flight which can be obstructed by rolling terrain or non-centrally available takeoff locations.

We preprogrammed aerial transects using Map Pilot for DJI on iOS flight software (hereafter Map Pilot).[10] Using the Map Pilot software, we included an altitude adjustment along each aerial transect using a 1-arc-second digital elevation model[11] such that the aircraft's altitude remained approximately constant at 120 meters above ground level in order to maintain consistent ground sampling distance (centimeters on the ground per pixel) in the imagery. Ground sampling distance was approximately 5 cm/px for the Zenmuse X3 RGB camera and approximately 8 cm/px for the RedEdge3 multispectral camera. For this analysis, we dropped 4 sites whose imagery was of insufficient quality to process.

Structure from motion (SfM) processing requires highly overlapping images, especially in densely vegetated areas.[12] We planned transects with 90% forward overlap and 90% side overlap at 100 meters below the lens. Thus, with flights being at 120 meters above ground level, we achieved slightly higher than 90/90% overlap for objects under 20 meters tall (91.6/91.6% overlap at the ground). Overlap values were based on focal length (3.6mm), sensor width (6.2mm), and image dimension (4000x3000 pixels) parameters of the Zenmuse X3 camera. Images were captured at a constant rate of 1 image every 2 seconds for both cameras. A forward overlap of 90% at 100 meters translates to a flight speed of approximately 6.45 m/s and a side overlap of 90% at 100 meters translates to transects approximately 17.2 meters apart. The RedEdge3 camera has a different focal length (5.4mm), sensor width (4.8mm), and image dimension (1280x960 pixels), which translates to image overlap of 80.7/80.7 % at 100m below the lens and 83.9/83.9 % at ground level. Approximately 1900 photos were captured over each 40 hectare survey area for each camera.

Structure from Motion (SfM) processing

We used structure from motion (SfM) to generate dense point clouds (Supplementary Figure 7), digital surface models (Supplementary Figure 9), and orthorectified reflectance maps (Supplementary Figure 8) for each field site.[12] We used Pix4Dmapper Cloud to process imagery using parameters ideal for images of a densely vegetated area taken by a multispectral camera. For 29 sites, we processed the RedEdge3 multispectral imagery alone. For three sites, we processed the RGB and the multispectral imagery in the same project to enhance the point density of the resulting point cloud. All SfM projects resulted in a single processing "block," indicating that all images in the project were optimized and processed together.

Creating canopy height models

We classified each survey area's dense point cloud into "ground" and "non-ground" points using a cloth simulation filter algorithm[2] implemented in the `lidR`[4] package. We rasterized the ground points using the `raster` package[13] to create a digital terrain model (Supplementary Figure 11) representing the ground underneath the vegetation at 1 meter resolution. We created a canopy height model (Supplementary Figure 12) by subtracting the digital terrain model from the digital surface model created in Pix4Dmapper.

Tree detection

We tested a total of 7 automatic tree detection algorithms and a total of 177 parameter sets on the canopy height model or the dense point cloud to locate trees within each site (Table 1 from main text). We used 3 parameter sets of a variable window filter using the `vwf()` function in the `ForestTools`[6] `R` package, including the default `winFun` parameter for the `vwf()` function as well as the "pines" and "combined" functions from 14 as the `winFun` parameter. We used 6 parameter sets of a local maximum filter implemented in `lidR`. We used 131 parameter sets of the algorithm from 15, which operates on the original point cloud. These parameter sets included those from 16 and 17. We used 3 parameter sets of the `watershed` algorithm implemented in `lidR`, which is a wrapper for a function in the `EBImage` package.[18] We used 3 parameter sets of `ptrees`[19] implemented in `lidR`[4] and `lidRplugins`[20] and which operates on the raw point cloud, without first normalizing it to height above ground level (i.e., subtracting the ground elevation from the dense point cloud). We used the default parameter set of the `multichm`[21] algorithm implemented in `lidR`[4] and `lidRplugins`.[20] Finally, we used 30 parameter sets of the experimental algorithm `lmfx`.[20]

Map ground data

Each orthorectified reflectance map was inspected to locate the 5 orange "X"s marking the center of the field plots (Supplementary Figure 6), though some plot centers were obscured due to dense interlocking tree crowns or because a plot center was located directly under a single tree crown. We were able to locate 110 out of 180 field plots and were then able to use these plots for validation of automated tree detection algorithms. We used the `sf` package[22] to convert distance-from-center and azimuth measurements of each tree in the ground plots to an x-y position on the SfM-derived reflectance map using the x-y position of the orange X visible in the reflectance map as the center.

Correspondence of automatic tree detection with ground data

We calculated 7 forest structure metrics for each field plot using the ground data collected by 1: total number of trees, number of trees greater than 15 meters, mean height of trees, 25th percentile tree height, 75th percentile tree height, mean distance to nearest tree neighbor, mean distance to 2nd nearest neighbor.

For each tree detection algorithm and parameter set described above, we calculated the same set of 7 structure metrics within the footprint of the validation field plots. We calculated the Pearson's correlation and root mean square error (RMSE) between the ground data and the aerial data for each of the 7 structure metrics for each of the 177 automatic tree detection algorithms/parameter sets.

For each algorithm and parameter set, we calculated its performance relative to other algorithms as whether its Pearson's correlation was within 5% of the highest Pearson's correlation as well as whether its RMSE was within 5% of the lowest RMSE. For each algorithm/parameter set, we summed the number of forest structure metrics for which it reached these 5% thresholds. For automatically detecting trees across the whole study, we selected the algorithm/parameter set that performed well across the most number of forest metrics (Supplementary Figure 14).

Segmentation of crowns

We delineated individual tree crowns with a marker controlled watershed segmentation algorithm[5] using the detected treetops as markers implemented in the `ForestTools` package.[6] If the automatic segmentation algorithm failed to generate a crown segment for a detected tree (e.g., often snags with a very small crown footprint), a circular crown was generated with a radius of 0.5 meters. If the segmentation generated multiple polygons for a single detected tree, only the polygon containing the detected tree was retained (Supplementary Figure 15). Image overlap decreases near the edges of the overall flight path, which reduces the quality of the SfM processing in those areas. Thus, we excluded segmented crowns within 35 meters of the edge of the survey area. Given the narrower field of view of the RedEdge3 multispectral camera versus the X3 RGB camera whose optical parameters were used to define the ~40 hectare survey area around each site, as well as the 35 meter additional buffering, the survey area at each site was approximately 30 hectares (Supplementary Table 1).

We used the `velox` package[23] to extract all the pixel values from the orthorectified reflectance map for each of the 5 narrow bands within each segmented crown polygon. Per pixel, we additionally calculated the normalized difference vegetation index,[3] the normalized difference red edge,[24] the red-green index,[25] the red edge chlorophyll index,[26] and the green chlorophyll index.[26] For each crown polygon, we calculated the mean

value for each raw and derived reflectance band (5 raw; 5 derived).

Classification of trees

We overlaid the segmented crowns on the reflectance maps from 20 sites spanning the latitudinal and elevation gradient in the study. Using QGIS, we hand classified 564 trees as live/dead (Supplementary Figure 16) and as one of 5 dominant species in the study area (*Pinus ponderosa*, *Pinus lambertiana*, *Abies concolor*, *Calocedrus decurrens*, or *Quercus kelloggi*) using the mapped ground data as a guide. We treated all trees classified as ponderosa pine as a "host" tree and all other species as "non-host" trees (Supplementary Figure 17).

We used all 10 mean values of the reflectance bands for each tree crown polygon to predict whether the hand classified trees were alive or dead using a boosted logistic regression model implemented in the `caret` package (accuracy of live/dead classification on a withheld test dataset: 96.4%).[27] For just the living trees, we similarly used all 10 reflectance values to predict the tree species using regularized discriminant analysis implemented in the `caret` package (accuracy of species classification on a withheld testing dataset: 64.1%; accuracy of WPB host/non-WPB-host (i.e., ponderosa pine versus other tree species) on a withheld testing dataset: 71.8%).

Finally, we used these models to classify all tree crowns in the data set as alive or dead as well as the species of living trees.

Calibrating heights of live and dead trees

We calibrated the live/dead classified tree heights (a Level3b product) by identifying 451 field-measured trees in the drone-derived tree data. Compared to field-measured live trees, drone-measured live tree height showed a small overestimation in height across the range of drone-measured tree heights (Supplementary Figure 18). The heights of dead trees greater than 20 m (as measured by the drone) showed a small underestimation of height, but heights of dead trees less than 20 m (as measured by the drone) showed a greater average underestimation (and variability) compared to the field measurements (Supplementary Figure 18).

Using the field and drone matched tree data for drone-measured trees greater than 20 m in height, we applied a conservative height correction to all live and dead trees in the Level 3b data product based on a linear regression of the difference between the field- and drone-measured tree heights as a function of drone-measured tree height:

$$\Delta_{height} = \beta_0 + X_{height}\beta_1 + X_{live}\beta_2 + X_{height}X_{live}\beta_3 + \epsilon(1)$$

where Δ_{height} represents the field-measured tree height subtracted from the drone-measured tree height, X_{height} represents the drone-measured tree height, and X_{live} represents a boolean variable for whether the tree is alive or dead. We applied this Δ_{height} to the drone-measured heights of each of the detected and classified trees in the dataset based on whether the tree was classified as live or dead and based on its drone-measured tree height (Supplementary Figure 19). The effect of the calibration of the tree heights across the range of site-level CWD is depicted in Supplementary Figure 20.

Rasterizing individual tree data

Because the tree detection algorithms were validated against ground data at the plot level, we rasterized the classified trees at a spatial resolution similar to that of the ground plots (Supplementary Figure 21). That is, we rasterized the individual tree data to 20 x 20 m pixels equaling 400 m^2, and the circular ground plots with 11.35m radius covered 404 m^2. In each raster cell, we calculated the: number of live trees, number of dead trees, number of ponderosa pine trees, total number of trees (of all species, including ponderosa pine), quadratic mean diameter (QMD) of ponderosa pine trees, and QMD of all trees of any species (overall QMD). We converted the count of ponderosa pine trees and the total tree count to a density measurement of trees per hectare (tpha) by multiplying the counts in each 20 x 20 m cell by 25 to create a "host density" and an "overall density" variable per cell.

Basal area consequences of proportion of dead host trees

The proportion of basal area killed is also an important measure of forest disturbance severity. We graphically assessed how the proportion of basal area killed varied as a function of proportion of host trees killed (i.e., the response variable of our formal statistical analysis). We opted to not treat proportion of basal area killed as a response variable in a second formal modeling framework due to unsatisfactory model fitting, likely arising from the additional uncertainty in estimates of basal area from allometric relationships with drone-measured tree height and/or the height calibrations depending on live/dead status prior to estimating each tree's basal area.

Supplementary References

1. Fettig, C. J., Mortenson, L. A., Bulaon, B. M. & Foulk, P. B. Tree mortality following drought in the central and southern Sierra Nevada, California, U.S. *Forest Ecology and Management* **432**, 164–178 (2019).

2. Zhang, W. *et al.* An easy-to-use airborne LiDAR data filtering method based on cloth simulation. *Remote Sensing* **8**, 501 (2016).

3. Rouse, W., Haas, R. H., Deering, W. & Schell, J. A. *Monitoring the vernal advancement and retrogradation (green wave effect) of natural vegetation.* (1973).

4. Roussel, J.-R., Auty, D., De Boissieu, F. & Meador, A. S. *lidR: Airborne LiDAR data manipulation and visualization for forestry applications.* (2019).

5. Meyer, F. & Beucher, S. Morphological segmentation. *Journal of Visual Communication and Image Representation* **1**, 21–46 (1990).

6. Plowright, A. *ForestTools: Analyzing remotely sensed forest data.* (2018).

7. DJI. Zenmuse X3 - Creativity Unleashed. *DJI Official* https://www.dji.com/zenmuse-x3/info (2015).

8. Micasense. MicaSense. https://support.micasense.com/hc/en-us/articles/215261448-RedEdge-User-Manual-PDF-Download- (2015).

9. DJI. DJI - The World Leader in Camera Drones/Quadcopters for Aerial Photography. *DJI Official* https://www.dji.com/matrice100/info (2015).

10. DronesMadeEasy. Map Pilot for DJI on iOS. *App Store* https://itunes.apple.com/us/app/map-pilot-for-dji/id1014765000?mt=8 (2018).

11. Farr, T. G. *et al.* The shuttle radar topography mission. *Reviews of Geophysics* **45**, (2007).

12. Frey, J., Kovach, K., Stemmler, S. & Koch, B. UAV photogrammetry of forests as a vulnerable process. A sensitivity analysis for a structure from motion RGB-image pipeline. *Remote Sensing* **10**, 912 (2018).

13. Hijmans, R. J. *et al. Raster: Geographic data analysis and modeling.* (2019).

14. Popescu, S. C. & Wynne, R. H. Seeing the trees in the forest: Using LiDAR and multispectral data fusion with local filtering and variable window size for estimating tree height. *Photogrammetric Engineering and Remote Sensing* 16 (2004).

15. Li, W., Guo, Q., Jakubowski, M. K. & Kelly, M. A new method for segmenting individual trees from the LiDAR point cloud. *Photogrammetric Engineering & Remote Sensing* **78**, 75–84 (2012).

16. Shin, P., Sankey, T., Moore, M. & Thode, A. Evaluating unmanned aerial vehicle images for estimating forest canopy fuels in a ponderosa pine stand. *Remote Sensing* **10**, 1266 (2018).

17. Jakubowski, M. K., Li, W., Guo, Q. & Kelly, M. Delineating individual trees from LiDAR data: A comparison of vector- and raster-based segmentation approaches. *Remote Sensing* **5**, 4163–4186 (2013).

18. Pau, G., Fuchs, F., Sklyar, O., Boutros, M. & Huber, W. EBImage: An R package for image processing with applications to cellular phenotypes. *Bioinformatics* **26**, 979–981 (2010).

19. Vega, C. *et al.* PTrees: A point-based approach to forest tree extraction from LiDAR data. *International Journal of Applied Earth Observation and Geoinformation* **33**, 98–108 (2014).

20. Roussel, J.-R. *lidRplugins: Extra functions and algorithms for lidR package.* (2019).

21. Eysn, L. *et al.* A benchmark of LiDAR-based single tree detection methods using heterogeneous forest data from the alpine space. *Forests* **6**, 1721–1747 (2015).

22. Pebesma, E. *et al.* *Sf: Simple features for R.* (2019).

23. Hunziker, P. *Velox: Fast raster manipulation and extraction.* (2017).

24. Gitelson, A. & Merzlyak, M. N. Spectral reflectance changes associated with autumn senescence of *Aesculus hippocastanum* L. And *Acer platanoides* L. Leaves. Spectral features and relation to chlorophyll estimation. *Journal of Plant Physiology* **143**, 286–292 (1994).

25. Coops, N. C., Johnson, M., Wulder, M. A. & White, J. C. Assessment of QuickBird high spatial resolution imagery to detect red attack damage due to mountain pine beetle infestation. *Remote Sensing of Environment* **103**, 67–80 (2006).

26. Clevers, J. G. P. W. & Gitelson, A. A. Remote estimation of crop and grass chlorophyll and nitrogen content using red-edge bands on Sentinel-2 and -3. *International Journal of Applied Earth Observation and Geoinformation* **23**, 344–351 (2013).

27. Kuhn, M. Building predictive models in R using the caret package. *Journal of Statistical Software* **28**, 1–26 (2008).
